# Intimate partner sexual violence during pregnancy and its associated factors in Northwest Ethiopian women

**DOI:** 10.3389/fsoc.2023.797098

**Published:** 2023-03-09

**Authors:** Zelalem Nigussie Azene, Mehari Woldemariam Merid, Asefa Adimasu Taddese, Zewudu Andualem, Nakachew Sewnet Amare, Birhan Tsegaw Taye

**Affiliations:** ^1^Department of Women's and Family Health, School of Midwifery, College of Medicine and Health Sciences, University of Gondar, Gondar, Ethiopia; ^2^Department of Epidemiology and Biostatistics, Institute of Public Health, College of Medicine and Health Sciences, University of Gondar, Gondar, Ethiopia; ^3^Department of Environmental and Occupational Health and Safety, Institute of Public Health, College of Medicine and Health Sciences, University of Gondar, Gondar, Ethiopia; ^4^School of Nursing and Midwifery, Asrat Woldeyes Health Science Campus, Debre Berhan University, Debre Berhan, Ethiopia

**Keywords:** sexual violence, pregnant women, intimate partner, Ethiopia, sexual and reproductive health

## Abstract

**Background:**

Violence against women is a global problem. In pregnant women, it is a particular concern as a virtue of the additional risks to the unborn child. Of different acts of violence, sexual violence shares the major contribution that results in short and long-term physical, sexual, reproductive, and mental health problems of pregnant women. Little is known about sexual violence during pregnancy in Ethiopia.

**Objective:**

this study aimed to assess the proportion and factors associated with intimate partners' sexual violence against pregnant women in Northwest Ethiopia.

**Methods:**

A cross-sectional study was conducted among 409 pregnant women in Debre Markos town from March to April 2018. The study participants were selected using a systematic random sampling technique. A pre-tested and validated questionnaire was used. Binary logistic regression analyses were done to identify associated factors and the adjusted odds ratio (AOR) with its 95 % confidence interval (CI) at a *p*-value of <0.05 was used to declare a significant association.

**Result:**

Of 409 pregnant women, 19.8% have experienced sexual violence by their intimate partner during their current pregnancy. Accordingly, the major intimate partner sexual violence during pregnancy was having unwanted sexual intercourse due to fear from the partner (13.4%), being forced to do something sexual that is degrading or humiliating (13.0%), and being physically forced to have sexual intercourse (9.8%). Living with her partner/husband (AOR: 3.73, 95% CI: 1.30, 10.69), uneducated educational status of partner (AOR: 2.43, 95% CI: 1.06, 5.56), and frequency of alcohol consumption (AOR: 3.20, 95% CI: 1.24, 8.26) were factors associated with increased occurrence of intimate partner sexual violence during pregnancy.

**Conclusion:**

The proportion of sexual violence against pregnant women by their intimate partner(s) was found to be common in our study. Socio-demographic and behavioral-related factors were risk factors for sexual violence. As a result, preventive strategies and interventions centering on the empowerment of those facing the greatest barriers to reproductive freedom require a shift from traditional ways of thinking.

## Introduction

Intimate partner sexual violence (IPSV) can occur in all types of intimate relationships regardless of gender identity or sexual orientation and it is not only defined by gender or sexuality but also by abusive behavior (Krug et al., 2002; Taylor and Gaskin-Laniyan, 2007; Zinzow et al., 2010; US Department of Justice, 2012; Abebe Abate et al., 2016). It is correspondingly defined as an act that results in any suffering or harm to women, including threats and deprivation of liberty, occurring either in public or private life committed by acquaintances or strangers (Karaoglu et al., 2005).

Globally, an estimated 35% of women have experienced either physical and/or IPSV (Kilonzo et al., 2009; Global WHO, 2013; Thomson, 2019) 9.12, and among 70% of survivors who were victimized by someone they knew, about 25% are sexually abused by an intimate partner or spouse (Centers for Disease Control and Prevention, 2014). Despite, sexual violence legislation in Africa, particularly, sub-Saharan Africa has been increasing (Nasir and Hyder, 2003; Angela, 2012) and the problem is preventable (ACOG, 2019), the occurrence ranged from 4 to 54% (Centers for Disease Control and Prevention, 2003; Head and Milton, 2014) 11. The magnitude of IPV ranged from worldwide (9), and 2-57% in Africa (10). In Ethiopia, the pooled prevalence of IPV was 26.1% in 2018 (11). As of evidence, approximately 15–71% of people experienced physical, sexual, or some combination of these types of violence at some point in their lives (12).

Violating pregnant mothers is a major public health crisis and a barrier to the development of the country (Angela, 2012; ACOG, 2019). As evidence indicated that victims of violence experience physical injury, mental health problems, and physical problems including suicide attempts, cardiovascular disease, unwanted pregnancy, registering late for prenatal care, suffering from preterm labor or miscarriage, or giving birth to low birth weight infants, gynecologic disease and substance abuse, which can all lead to hospitalization, disability or death (Savona-Ventura, 2001; Centers for Disease Control and Prevention, 2003; Karaoglu et al., 2005; Head and Milton, 2014; ACOG, 2019). Sexually violated women are more likely to have a poor quality of health as compared to women without a history of abuse (Bonomi and Rivara, 2007; Pikarinen et al., 2007; Ellsberg et al., 2008; Zinzow et al., 2011) and develop complicated pregnancy outcomes (Stenson et al., 2003). Another report also showed that nausea and vomiting, tiredness, backache, heartburn, constipation, vaginal discharge, leg cramps, edema, headache, urinary incontinence, pelvic girdle relaxation, and urinary tract infections during pregnancy are results of sexual violence which changes the normal physiology (Freeman, 1980; Enkin and Neilson, 2000; Draper, 2006) and usually have no bearing on the outcome of pregnancy (Freeman, 1980; Enkin and Neilson, 2000).

Youn age, high-risk behavior including alcohol/substance misuse, and other forms of violence such as domestic violence by a spouse or partner in an intimate relationship against the other spouse or partner (Avegno and Mills, 2007; Kilpatrick et al., 2007; Tjaden and Thoennes, 2000). Other researchers also explained socio-economic, cultural, biological, and environmental factors. Thus, low income, low education status, involvement in aggressive or delinquent behavior as an adolescent, alcohol, and drug use, personality factors including low self-esteem, depression, antisocial personality disorders, having experienced violence as a child, gender differences in society, rigid gender roles and traditional norms that favor men, and most women are subordinate to their husbands and the acceptance of such behavior by society are some of the known related factors (Rosenberg and Hammond, 1998; World Health Organization Document, 2004). As a result, sexual violence causes serious physical, mental, sexual, and reproductive health problems for women in the short and long-term affecting their whole life.

Despite this considerable health burden, previous studies focused on the prevalence and determinants of intimate partner violence; so, little is recognized about the sexual violence of pregnant women in Ethiopia, particularly in the study area. Moreover, evidence is not well documented on which factors are contributed to an increase in intimate partner sexual violence in the course of pregnancy. Hence, this study aimed to assess the proportion and factors associated with intimate partner sexual violence in pregnant women.

## Methods

### Study design, period, and setting

A facility-based cross-sectional study was conducted from March 16 to April 14, 2018, in Debre Markos town, Northwest Ethiopia. It is located in the East Gojjam Zone, far 299 kilometers Northwest of Addis Ababa, the capital of Ethiopia. Depending on the population projection of Ethiopia for all regions at the woreda level from 2014–to 2017, the town has an estimated total population of 92,470, of which 46,738 were females. It also comprised one referral hospital, three public health Centers, seven private clinics, and 14 health posts, seven in rural and seven in urban areas. All four public health facilities and three private clinics in the town are providing antenatal care services.

### Source and study population

All pregnant women who visited the public health institutions in the town for ANC service were the source population, whereas pregnant women who were available during the data collection period in the selected institutions were the study population.

### Sample size determination and sampling procedure

The sample size (422 pregnant women) was computed by using a single population proportion formula by considering the following assumptions: The proportion of women who have experienced IPSV during pregnancy was 50%, the level of significance was 95%, a margin of error 5%, and non-response rate 10%. The sample size was allocated proportionally to the four health facilities in the town according to the number of previous client flow (pregnant women) that visited each health facility during the preceding month before data collection. Then, the study participants were selected through a systematic random sampling technique.

### Study variables and measurements

IPSV during pregnancy is a response variable, whereas socio-demographic, husband or partner characteristics, socio-cultural and family experience of violence, and reproductive variables were independent variables included in this study. An intimate partner was defined as a current spouse, co-habited (live in the same house without formal marriage), current non-marital partner (boyfriend), former partner, or spouse. Sexual violence was considered in this study if the study participant's response is “Yes” to any one of the items such as uses of force, coercion, or psychological intimidation to force the woman to engage in a sex act against her will whether or not it is completed.

### Data collection tools, procedures, and quality control

The content validity of the questionnaire was judged by a group of researchers who are experts on maternal and child health to evaluate and enhance the items in the question. A face-to-face interviewer-administered questionnaire (Devries et al., 2011) was used to collect data from all pregnant women who consented to be part of the study. To ensure the quality of data, the questionnaire was first developed in English, then translated into the local language (i.e., Amharic), and finally back into English to check its consistency by local and English language professionals. Five female midwives for data collection and one BSC midwife for supervision were recruited from each of the public health institutions in Debre Markos town. They were trained for one day on the objective of the study and the ways of data collection. Supervisors and principal investigators have closely monitored the day-to-day data collection process. Finally, data were sorted, checked, entered into the EPI-Info, and cleaned for analysis.

### Data processing and statistical analysis

The questionnaires were coded, and the data were entered and cleaned by EPI-Info 7.0 statistical software and then exported to SPSS version 20 for further analysis. Data were summarized and descriptive statistics were carried out. Model fitness was checked using Hosmer and Lemeshow test. Bivariable and multivariable logistics regressions were fitted to identify the significance of associations between the outcome and independent variables. In multivariable logistic regression analysis, a *p*-value of ≤0.05 with 95% CI for the AOR was used to determine the significant association.

## Results

### Maternal socio-demographic characteristics

Of a total of 422 sampled population, 409 participants were involved in this study making a response rate of 96.9%. The mean age of women was 27.1 ± SD 5.6 years. More than half (52.1%) of the respondents were in the age group of 17 to 26 years. The samples were predominantly urban (71.6%) and orthodox Christian 339 (82.9%) religion followers. Regarding the occupational status of the respondents, 46.0 % were housewives. About 95.6 % of the respondents were married and 31.3% have no formal education. Of the total participants, 35.3% of women reported that household decision was made by their husband only and 63.6% of participants had a joint decision with their husband (see Table 1).

**Table 1:** Socio-demographic characteristics of the study participants (*n* = 409).

**Characteristics**		**Frequency**	**Percentage**
Age of women in years	17–23	118	28.9
	24–26	95	23.2
	27–30	106	25.9
	31–46	90	22
Religion	Orthodox	339	82.9
	Muslim	59	14.4
	Protestant	8	2
	Catholic	3	0.7
Place of residence	Rural	116	28.4
Urban	293	71.6
Current marital status	Single	8	2
	Married	391	95.6
	Divorced	7	1.7
	Widowed	1	0.2
	Separated	2	0.5
Type of marriage ceremony	No ceremony Civil marriage Religious marriage Customary marriage	29 52 56 264	7.2 13 14 65.8
Women's education	No formal education	128	31.3
	Primary education	64	15.6
	Secondary education	103	25.2
	More than secondary	114	27.9
Partner's education	No formal educational Primary education Secondary education More than secondary	123 44 95 147	30.1 10.8 23.2 35.9
Women's occupation	Housewife	188	46
	Farmer	75	18.3
	Student	1	0.2
	Private employee	18	4.4
	Government employee	79	19.3
	Merchant	35	8.6
	Others^a^	13	3.2
Partner's occupation	Farmer Student Private employee Government employee Merchant Others^b^	107 3 93 122 66 18	26.2 0.7 22.7 29.8 16.2 4.4
The decision-maker in the household	Husband only Me only Jointly	132 4 238	35.3 1.1 63.6
Living with partner/husband	Yes No	35 374	8.6 91.4
Partner's unfaithful relation	Yes No	238 171	58.19 41.81
Partner's frequency of alcohol drinking	Daily 1–2 times per week ≤ 3 times per month	92 93 69	36.2 36.6 27.2
Monthly income in ETB	<2,500	212	51.8
	≥2,500	197	48.2

Others^a^, daily laborer, unemployed; Others^b^, Driver, Deacon, daily laborer, priest.

### Obstetrics characteristics of participants

Of the participants, 114 (28.4%) respondents got married before the age of 18 year-olds and 44 (10.8%) became pregnant for the first time. More than half (51.6%) start their ANC follow-up in the first trimester of pregnancy. Moreover, 24 (5.9%) of the study participants had a history of abortion (see Table 2).

**Table 2:** Obstetrics characteristics of participants (*n* = 409).

**Characteristics**		**Frequency**	**Percentage**
Age at first pregnancy	<18 years	44	10.8
	≥18 years	365	89.2
Age at first marriage	<18 years ≥18 years	114 287	28.4 71.6
Gravidness	Primigravida Multigravidas	194 215	47.4 52.6
Pregnancy desired by	Women Partner Both of us	144 148 117	35.29 36.1 28.61
First ANC initiation	1^st^ trimester 2^nd^ trimester 3^rd^ trimester	211 169 29	51.6 41.3 7.1
History of abortion	Yes	24	5.9
	No	385	94.1

### The proportion of IPSV among pregnant mothers

This indicated that the overall proportion of intimate partner sexual violence during the current pregnancy was 19.8% (95% CI: 15.9, 23.5). Thus, the major IPSV during pregnancy was having unwanted sexual intercourse due to fear from the partner 55 (13.4%), being forced to do something sexual that is degrading 53 (13.0%), and being physically forced to have sexual intercourse 40 (9.8) (see Table 3).

**Table 3:** Proportion of IPSV among pregnant women in Debre Markos town, Northwest, Ethiopia (*n* = 409).

**Violence items**	**Frequency**	**Percentage**
Physically forced you to have sexual intercourse	40	9.8
Having unwanted sexual intercourse because of fear from the partner	55	13.4
Forced you to do something sexual that is degrading or humiliating	53	13.0
The overall prevalence of IPSV during pregnancy	81	19.8

### Factors associated with IPSV among pregnant women

In the bi-variable analysis; extramarital sex, education status of partner, frequency of alcohol consumption, residence, living with partner/husband, the timing of ANC start, educational status of women, had children from another partner (s), partner's occupational status, and partner's unfaithful relation was significantly associated with sexual violence by an intimate partner during pregnancy but lost the significance in multivariable analyses. After controlling the possible confounders, however, only living with her partner/husband, education status of the partner, and frequency of alcohol consumption were found significantly associated with increased prevalence of IPSV during pregnancy.

In our study, women whose husbands/partners consume alcohol frequently were 3.2 times (AOR: 3.20, 95% CI: 1.24, 8.26) more likely to be victimized by intimate partner sexual violence than their counterparts. The educational status of the partner was also another sociodemographic variable predicting the likelihood of IPSV during the time of pregnancy. Pregnant women whose partners/husbands were uneducated were at 2.43 (AOR: 2.43, 95% CI: 1.06, 5.56) times higher risk to attempt IPSV as compared to their counterparts (see Table 4).

**Table 4:** Bivariable and Multivariable analysis of factors associated with IPSV among pregnant women in Debre Markos town, Northwest, Ethiopia (*n* = 409).

**Variables**	**Category**	**IPSV**		**COR (95% CI)**	**AOR (95% CI)**
		**Yes**	**No**		
Timing of ANC start	1^st^ trimester	33	178	0.58 (0.35–0.95)	0.72 (0.38–1.37)
	2^nd^ and 3^rd^ trimester	48	150	1(Ref)	
Residence	Rural	30	86	1.66 (0.99–2.77)	2.52 (0.76–8.28)
	Urban	51	242	1(Ref)	
Frequency of alcohol consumption	Daily	32	60	3.56 (1.56–8.09)	3.2 (1.24–8.26)^*^
	1–2 times per week	27	66	2.73 (1.19–6.26)	2.84 (1.11–7.26)^*^
	≤ 3 times per month	9	60	1(Ref)	
Women's education	Not educated	30	98	1.38 (0.83–2.29)	0.63 (0.27–1.5)
	Educated at least primary level	51	230	1(Ref)	
Partner's education	Not educated	37	86	2.37 (1.43–3.91)	2.43 (1.06–5.56)^*^
	Educated at least primary level	44	242	1(Ref)	
Extramarital sex	Yes	28	71	1(Ref)	
	No	53	257	0.52 (0.31–0.89)	0.67 (0.29–1.54)
Partner's occupation	Farmer	27	80	1(Ref)	
	Private employee	23	70	0.97 (0.51–1.85)	3.03 (0.81–11.39)
	Gov't employee	13	109	0.35 (0.17–0.73)	1.96 (0.43–8.95)
	Merchant	13	53	0.73 (0.34–1.53)	3.72 (0.81–17.12)
Had children from another partner (s)	Yes	20	43	2.17 (1.19–3.95)	1.43 (0.56–3.64)
	No	61	285	1(Ref)	
Living with partner/husband	Yes	13	22	2.66 (1.27–5.54)	3.73 (1.3–10.69)^*^
	No	68	306	1(Ref)	
Partner's unfaithful relation	Yes	59	179	1(Ref)	
	No	22	149	0.45 (0.26–0.77)	0.54 (0.27–1.06)

^*^p < 0.05, Hosmer and Lemeshow goodness of fit (*p* = 0.71).

Furthermore, living with her husband also affected the occurrence of sexual violence during pregnancy. The pregnant women who were living with their husbands were at 3.73 (AOR: 3.73, 95% CI: 1.30, 10.69) times increased risk of being abused by their intimate partner as compared to women living alone.

## Discussion

In Ethiopia, under the current sustainable development goal (SDG) period, the welfare of mothers, newborns, and children remains a top priority for health, but intimate partner sexual violence has become one of the main contributing factors that adversely affect the health of the woman and her fetus. To the best of our knowledge, this study is the first of its kind to exclusively quantify the proportion of IPSV during pregnancy along with the associated factors in Ethiopia. Hence, the current study has particularly assessed the proportion and the factors of IPSV in the setting.

Accordingly, the key findings of this study point out the role of social determinants of health in maternity services in Ethiopia and the necessity of conducting further research using a triangulated methodological approach to address deep-rooted social determinants of health in the context of IPSV. The overall proportion of IPSV during the current pregnancy was found to be 19.8% (95% CI: 15.9, 23.5). Thus, the major IPSV during pregnancy was having unwanted sexual intercourse because of fear from the partner (13.4%) and being forced to do something sexual that is degrading or humiliating (13.0%). Moreover, living with her spouse, not being educated educational status of her spouse, and frequency of alcohol consumption (drunk daily and 1–2 times per week) were important determinants associated with IPSV during pregnancy.

The result of our findings noted a higher prevalence of IPSV during the current pregnancy compared to different studies conducted in Tanzania (Sigalla, 2017), Rwanda (Ntaganira et al., 2008), Ghana (Ogum Alangea et al., 2018), Brazil (Puccia et al., 2018), and other regions of the country (Laelago et al., 2014; Yimer et al., 2014; Gebrezgi et al., 2017). There could be justified different perspectives. For instance, the community perception regarding intimate partner violence, violence measurement across scholars, and cultural differences among people across regions were some of the reasons for variation in the prevalence of intimate partner sexual violence. The probable cause of this situation might be due to the variations in culture, social norms, and the implementation of laws that prevent violence against women. For example, evidence from the 2016 EDHS has indicated that a lower proportion of males supporting wife-beating in southern Ethiopia at 14.9% as compared to the Harari region at 22.6% indicating that differences in social norms might contribute to the differences in proportions of IPSV. On the other hand, the current prevalence of IPSV was lower than in some other studies conducted elsewhere (Rosenberg and Hammond, 1998; Reed, 2015; Abebe Abate et al., 2016; Ayodapo et al., 2017; Field et al., 2018; Pengpid et al., 2018; Shamu, 2018; Lencha et al., 2019).

The disparity between our findings and across literature can be explained from different standpoints. Of these, it might be due to differences in study designs, such as the study conducted in Abay Chomen district, Oromia, Ethiopia was a community-based study whereas this study was facility-based which might have missed those women who were not coming to health facilities. It is also determined by the observed differences in cultural acceptability of domestic violence and hence underreporting and fear of disclosing the IPSV exposure (Stöckl et al., 2014). Furthermore, the majority of studies on violence during pregnancy measure only physical violence, but since our study was on sexual violence women usually do not report it while it is most detrimental to women's and their children's wellbeing. But, our finding was consistent with a few studies conducted somewhere (Tanimu et al., 2016; Malan et al., 2018; Ogum Alangea et al., 2018). Furthermore, health education and promotion interventions to reduce intimate partner sexual violence during pregnancy are recommended to facilitate and support improved use of skilled care during the maternal continuum of care, self-care, and home care practices for the woman and newborn. This also calls for an understanding of the joint responsibilities of men and women, so that they become equal partners in public and private lives and encouraging and enabling men to take responsibility for their sexual and reproductive behavior.

This study identified the factors associated with IPSV among pregnant women. Accordingly, women whose husbands or partners consume alcohol frequently were nearly three times more likely to be victimized by IPSV than their counterparts. This has been supplemented by different studies conducted so far (Laelago et al., 2014; Yimer et al., 2014; Tanimu et al., 2016; Gebrezgi et al., 2017; Alebel et al., 2018; Field et al., 2018; Ogum Alangea et al., 2018; Lencha et al., 2019). There could be different explanations for the risk of IPSV in women by their partners who are frequent alcohol consumers. For instance, alcohol drinking can cause the individual to develop an aggressive character, and misunderstanding verbal or non-verbal cues, and alcohol usage might be a source of dispute in relationships and thereby result in violent behavior (Stöckl et al., 2014; Jewkes et al., 2002). Moreover, some persons may intentionally use alcohol to hide behind the alcohol involved in antisocial behaviors such as violence against their partners.

Another factor significantly associated with intimate IPSV was unable to read and write the education level of the husband or partner. Thus, pregnant women whose partners/husbands had no education were at higher risk of sustaining sexual violence. This was in agreement with some studies conducted elsewhere (Abebe Abate et al., 2016; Bifftu et al., 2017; Gebrezgi et al., 2017; Alebel et al., 2018; Ogum Alangea et al., 2018). This can be explained that when partner education status increases the ability to negotiate may increase and as a result, the violence will decrease. In addition, it is a fact that partners with no formal education were more likely to have traditional perceptions regarding gender equality (Naved and Persson, 2008). Evidence revealed that low levels of education and lack of discussion to decide jointly with partners increase women's likelihood of experiencing violence during pregnancy (Abeya et al., 2011).

The authors acknowledged some limitations that should be considered when interpreting the results. One, the study was cross-sectional, a design that does not permit the establishment of cause-effect relationships. Secondly, due to the sensitive nature of the issue (sexual violence), women might feel fear to disclose their exposure (social desirability bias), resulting in an underestimation of the result. So, there is a need for further qualitative research to address behavioral factors.

## Conclusion

This result indicated that intimate partner sexual violence during pregnancy is a major social problem in Ethiopian women. The risk factors of IPSV during the pregnancy period were connected to social determinants of health. Therefore, it is recommended that an optimal intervention such as health education communication that discourages violence against women, provision of women-centered care, and advocacy/empowerment intervention should be implemented.

## Data availability statement

The original contributions presented in the study are included in the article/supplementary material, further inquiries can be directed to the corresponding author.

## Ethics statement

The studies involving human participants were reviewed and approved by University of Gondar, Ethiopia. The patients/participants provided their written informed consent to participate in this study.

## Author contributions

ZAz wrote the proposal, participated in data collection, and drafted the paper. MM, AT, ZAn, NA, and BT participated in data analysis, and manuscript preparation and revised the subsequent drafts of the paper. All the authors read and approved the final manuscript.
